# Repeated stereotactic radiotherapy of recurrent ventricular tachycardia

**DOI:** 10.1016/j.ctro.2023.100609

**Published:** 2023-03-01

**Authors:** Jakub Cvek, Lukáš Knybel, Josef Kautzner

**Affiliations:** Department of Oncology, University Hospital Ostrava, Ostrava, Czech Republic; Faculty of Medicine, University of Ostrava, Ostrava, Czech Republic; Department of Oncology, University Hospital Ostrava, Ostrava, Czech Republic; Faculty of Medicine, University of Ostrava, Ostrava, Czech Republic; Institute for Clinical and Experimental Medicine, Prague, Czech Republic

Dear Sir,

With interest, we have read recently the paper by Siklody and colleagues [1], describing single institution experience with the second session of stereotactic arrhythmia radioablation. We want to congratulate them on adding another piece to the puzzle of knowledge. However, it is important to emphasize that our group already published two papers dealing with re-do radioablation [2], [3]. One radiotherapy retreatment was due to the apparent inaccuracy of targeting the critical region of the arrhythmogenic substrate (2). The other paper summarized the experience with this case and the other two in whom re-do treatment was indicated for the specificity of the substrate (i.e., cardiac fibroma) or by extensive substrate (i.e., large septal scar in dilated cardiomyopathy) [3]. Therefore, we feel that we could contribute to the discussion.

We agree with optimizing the prescribed dose and target volume to achieve the optimal therapeutic response. However, in the case of cardiac radiotherapy, the optimization is more complex, and it is difficult to avoid occasional treatment failure for several reasons. The authors use rather small volumes and doses compared to other centres, which makes the comparison of efficacy and safety problematic. Although one can assume lower presented coverage of Planning Target Volume (PTV) by the prescribed dose as authors respected the 12 Gy tolerance limit for the coronary arteries, it would be interesting to compare the intersection of primary and secondary SBRT PTVs with our results [3]. The localization of the PTVs is nicely visible from the presented figures, however, it is not clear why the secondary PTV (9.69 ml) is shown as larger volume than the primary one (25.54 ml). Perhaps, the use of the Sørensen-Dice index would be helpful to measure the similarity between the two sets of data. Alternatively, or in addition, the Hausdorff distance might be used. The other important factor is deciding how much the substrate should be modified. The authors claim that they targeted only clinical VT; however, in one of our re-do cases, we did have several clinical VTs that required more extensive coverage of the substrate and repeated radiotherapy. One can also agree with the importance of accurate registration of electroanatomical maps and simulation CT [2]. However, our experience shows that even when using a sophisticated strategy of merging data, it is not possible to achieve a complete 100% match [4]. We can confirm that the use of ICD tracking allows for lower volumes. However, we are unsure whether the available technologies allow us to capture complex cardiac movements [5] as the authors declare. Furthermore, the controversy over the definition of small target volumes also has been published [6].

A challenge remains to minimize the dose burden to surrounding organs such as the stomach or esophagus, and especially the heart substructures, where precise dose-volume constraints still need to be discovered. Preclinical data suggest the possibility of a prescribed dose interval of 20–35 Gy. Here the authors declare a cumulative dose of 35 Gy needed to homogenize the substrate, and although it is unclear in what volumes this was achieved in both cases (Dmax for the first case 31.09 Gy and 32.63 Gy), the results presented show a promise. The description of the dose of 15 Gy in the transitory region and the documentation of slowing VTs after SBRT, is also interesting and corresponds with our results [2], [3].

Finally, the authors correctly point out that the data on late toxicities of radiotherapy is limited. In this respect, we recently published a case of oesophagopericardial fistula [7] and observed other cases of progression of valvular heart disease, requiring an intervention [8]. The authors also observed a case of progression of aortic valve disease.

The clinical use of radiosurgery for the ablation of refractory VT has been known since 2014. However, the clinical experience is still very limited as a relatively small number of patients have been treated worldwide. Furthermore, repeated procedures have been published even less frequently, reinforcing the importance of international collaborations such as STOPSTORM [9] to learn about the benefits and risks of this relatively new method as soon as possible.

## Declaration of Competing Interest

The authors declare the following financial interests/personal relationships which may be considered as potential competing interests: JC reports personal fees from Accuray, and Roche for lectures. JK reports personal fees from Abbott-St Jude Medical, Bayer, Biosense Webster, Biotronik, Boston Scientific, Cath Vision, Medtronic, Boehringer Ingelheim, Pfizer, and ProMedCS for lectures, advisory boards, and consultancy. JC, JK, and LK report grants from Ministry of Health, Czech Republic and European Commission H2020.
